# RETRACTION: Microrna‐379 Suppresses Osteosarcoma Progression by Targeting PDK1

**DOI:** 10.1111/jcmm.18567

**Published:** 2024-08-07

## Abstract

**RETRACTION:** Li, Z., Shen, J., Chan, M. T. V., and Wu, W. K. K. (2017). Microrna‐379 Suppresses Osteosarcoma Progression by Targeting PDK1. *Journal of Cellular and Molecular Medicine*, 21(2), 315–323. https://doi.org/10.1111/jcmm.12966

The above article, published online on 25 October 2016 in Wiley Online Library (wileyonlinelibrary.com), has been retracted by agreement between the journal Editor‐in‐Chief, Stefan Constantinescu; The Foundation for Cellular and Molecular Medicine and John Wiley and Sons Ltd. The retraction has been agreed upon following an investigation into concerns raised by a third party, which revealed inappropriate duplications of image panels (Figures 2D, 3, 4G, 6A and F) within the article and between this and other articles that were previously published or published shortly after in a different scientific context. The authors were unable to provide a satisfactory explanation and the provided raw data could not explain the identified issues, which has led the editors to lose confidence in the data presented. Therefore, the editors consider the conclusions substantially compromised and are retracting the paper. The co‐author, J. Shen stated that they were not involved in this research and had no knowledge about its publication, and their correspondence email address was added without their consent. The authors, including Z. Li who submitted the manuscript, were informed of the decision to retract but did not respond to the retraction or the retraction wording.

**RETRACTION:** Li, Z., Shen, J., Chan, M. T. V., and Wu, W. K. K. (2017). Microrna‐379 Suppresses Osteosarcoma Progression by Targeting PDK1. *Journal of Cellular and Molecular Medicine*, 21(2), 315–323. https://doi.org/10.1111/jcmm.12966

The above article, published online on 25 October 2016 in Wiley Online Library (wileyonlinelibrary.com), has been retracted by agreement between the journal Editor‐in‐Chief, Stefan Constantinescu; The Foundation for Cellular and Molecular Medicine and John Wiley and Sons Ltd. The retraction has been agreed upon following an investigation into concerns raised by a third party, which revealed inappropriate duplications of image panels (Figures 2D, 3, 4G, 6A and F) within the article and between this and other articles that were previously published or published shortly after in a different scientific context. The authors were unable to provide a satisfactory explanation and the provided raw data could not explain the identified issues, which has led the editors to lose confidence in the data presented. Therefore, the editors consider the conclusions substantially compromised and are retracting the paper. The co‐author, J. Shen stated that they were not involved in this research and had no knowledge about its publication, and their correspondence email address was added without their consent. The authors, including Z. Li who submitted the manuscript, were informed of the decision to retract but did not respond to the retraction or the retraction wording.

